# Cross-National Variations in Scientific Ethics: Exploring Ethical Perspectives Among Scientists in China, the US, and the UK

**DOI:** 10.1007/s11948-024-00505-0

**Published:** 2024-09-11

**Authors:** Di Di, Elaine Howard Ecklund

**Affiliations:** 1 Department of Sociology, Santa Clara University, 500 El Camino Real, Santa Clara, CA 95053 USA; 2 Department of Sociology, Rice University, 6100 Main Street, Houston, TX 77005 USA

**Keywords:** Science ethics, Physics, Academic responsibilities, Cross-national comparison

## Abstract

This research explores the perspectives of academic physicists from three national contexts concerning their roles and responsibilities within the realm of science. Using a dataset comprised of 211 interviews with scientists working in China, the United States, and the United Kingdom, the study seeks to explain whether and in what manner physicists conceptualize scientific ethics within a global or national framework. The empirical findings bring to light disparities across nations in the physicists’ perceptions of what constitutes responsible mentorship and engagement in public service. These cross-national variations underscore the moral agency of physicists as they navigate the ethical standards embraced by the global scientific community vis-à-vis those that are specific to their respective national contexts. The study’s empirical insights may carry significant implications for both policymakers and ethicists, underscoring the imperative of soliciting and acknowledging the perspectives of academic scientists working and living in disparate national contexts when formulating comprehensive science ethics frameworks. Such inclusive and context-aware approaches to shaping ethics in science can contribute to the cultivation of a more robust and universally relevant ethical foundation for the scientific community.

## Introduction

International collaborations are a common practice among academic scientists (Wagner & Leydesdorff, 2009), and such collaborations frequently require universal ethical principles with ethical considerations specific to their context (Wang & Yan, 2019). To offer implications for how academic scientists adopt and reconcile universal ethical principles and the ethical considerations specific to their context, this research focuses on physicists situated in the United States (US), the United Kingdom (UK), and the People’s Republic of China (PRC) and their ethical perspectives. The primary objective is to address two fundamental research questions: (1) What are the perceived ethical obligations held by physicists, and (2) what similarities and disparities in these perceptions exist across nations? The study acknowledges the limitations of interview data in representing generalizable trends but maintains that the exploratory nature of interviews is a suitable method for investigating physicists’ ethical perspectives (Maxwell, 2004). Additionally, interview data can inform the development of instruments for larger-scale studies.

Drawing on previous research (Bird, 1994; Pimple, 2002), this study anticipates that scientists will speak about their research, teaching, and social responsibilities in their discussions of science ethics. This research, however, seeks to uncover whether and how cross-national differences manifest in these reflections, shedding light on how academic scientists navigate between global research ethics and context-specific norms.

## The Scientist’s Responsibilities

The duties and responsibilities of scientists, especially in international collaborations, include sharing ideas and resources, which contribute to establishing universal ethical principles in scientific research (Phillips et al., 2020). Nevertheless, ethical violations persist (Price, 2006). A survey of biostatisticians in more than 20 national contexts reported that more than half of the respondents had heard of fraudulent research (Ranstam et al., 2000). A meta-analysis in the US, the UK, and Australia further revealed that only 1.97% of scientists had been involved in fabrication, falsification, or plagiarism, but 33.7% admitted to other ethically problematic research practices (Fanelli, 2009). The persistence of ethical violations underscores the importance of being aware of and discussing research ethics among scientists (De Vries et al., 2006).

In addition to an awareness of research ethics, scientists often say that research should benefit society, although their interpretations of this vary (Frankel, 2015). For example, some scientists advocate for politically neutral research topics (Reiser & Bulger, 1997), while others debate the responsibility for the potential misuse of their discoveries (Corley et al., 2016). Still others stress the importance of communicating their work to the public (Liu, 2011) or express the need for better tools for scientific outreach (Devonshire & Hathway, 2014).

The significance of teaching and mentoring, often overlooked in scholarly discussions of science ethics, cannot be understated in the cultivation of a thriving scientific community (Bird, 1994), particularly in nations with limited resources in developing science infrastructure (Hamer et al., 2019). Research in the US further demonstrates that mentoring about ethics plays a pivotal role in mitigating unethical behavior among junior scientists (Anderson et al., 2007).

Although discussions about scientific ethics transcend international boundaries and encompass aspects of research, teaching, and mentoring, variations exist among different nations. There is an ongoing debate concerning the applicability of universal ethical standards, such as obtaining informed consent from research participants in certain countries (Benatar, 2002). Therefore, it is important to examine context-specific dialogues on the ethics of science to contextualize the scientists’ perspectives within the unique dynamics of their respective working environments.

## Discourse on the Contextual Responsibilities of Scientists

Each of the countries in this study offers both shared and distinct perspectives on science ethics. Historical perspectives on US science contend that scientists involved in basic science are not responsible for the consequences of their work, although they do have a responsibility to contribute to society (Nielsen, 1955). Recent discussions on science ethics in the US, however, have encouraged scientists to reflect on a broader notion of the ethical implications of their research (Relman, 2013), identify potential risks and harms (Reiser & Bulger, 1997), and consider whether their work serves the broader domain of “justice” (Mamo & Fishman, 2013). Some social scientists and ethicists also propose engagement in advisory boards and scientific outreach (Andrews et al., 2005; Ecklund et al., 2012).

In the UK, there are similar debates on the extent and nature of academic scientists’ social responsibilities (Polanyi, 1946; Wolpert, 2005). While some separate science ethics from tech ethics (Wolpert, 2005), others argue that science and its societal implications are inseparable (Calvert & Martin, 2009). Scientists in the European Union, for instance, are encouraged to consider potential harms in their research (Amodio et al., 2021).

In both the US and the UK, discussions on science ethics revolve around the balance between research autonomy and social responsibilities (Calvert & Martin, 2009; Relman, 2013). In contrast, China’s ethical discourse is influenced by a blend of what Wang and Yan (2019 p. 1726) call a “traditional Chinese approach to ethics” and “Western thinking” (Jing & Doorn, 2020). Scholars argue that ethical principles in academic science in China are primarily borrowed from the ethical discourse in Western societies, such as the US and the UK, which were inspired by Western philosophies, such as deontological ethics (Alexander & Moore, 2021). However, Confucianism introduces unique ethical considerations that may not align with Western perspectives (Jing & Doorn, 2020). For example, Confucianism does not mention where a person’s responsibility ends whereas science ethicists in the US and the UK try to develop a clear list about the traits of responsible scientists (Jing & Doorn, 2020).

What remains unclear is how the national-level discourse of science ethics influences scientists’ ethical perspectives. The present study focuses on the ethical perspectives of academic physicists relying on interviews.

## Data and Methods

The data for the present study was derived from a broader research project that focuses on how physicists in China, the US, and the UK perceive science ethics. These countries were selected due to their distinct position in the core networks of scientific collaborations and their substantial international collaborations. While the US and the UK have long been situated in the core network of scientific collaborations (Leydesdorff & Wagner, 2008b), the expansion of the Chinese government’s investment in scientific research, beginning in the early 2000s, made China a new player in international collaboration (Leydesdorff & Zhou, 2005). Today, the US and the UK have the most bilateral research collaborations with China, measured by the number of publications (Adams et al., 2022).

From a methodological standpoint (Hantrais, 1999), the differences, similarities, and interconnections in the scientific communities of these countries provide a unique opportunity to identify both prominent and subtle cross-national distinctions in the physicists’ ethical perspectives. However, it is crucial to acknowledge that comparisons between these countries may not fully represent the global scientific community, as discussed in the forthcoming sections.

For the sampling strategy, the study relied on the National Research Council (NRC) in the US to identify physics departments with varying levels of research productivity. This approach led to the selection of nine universities, ensuring representation from both private and public institutions across different geographical locations. In the UK, a list of seven elite and non-elite universities was compiled based on research productivity data from the Web of Science (WOS), insider opinions, and public rankings. The final sampling frame consisted of 179 potential respondents in the US and 132 in the UK. Among them, 90 physicists in the US and 71 in the UK participated in interviews. To address the over-representation of physicists from elite universities in the UK, the researchers conducted ten additional interviews with physicists from non-elite universities, resulting in a final sample of 81 UK physicists.

Table 1 presents the demographic characteristics of the respondents included in the final sample. In terms of the interview modality, more than 40% of interviews in the US and 50% in the UK were conducted in person, typically in the respondents’ offices or other locations of their choice. The remaining interviews were carried out via phone or video conferencing. Research has shown that there are minimal differences in the quality of in-person, telephone, and video-conference interviews (Johnson et al., 2019).

**Table 1:** Respondents demographics

	China		US		UK	
	N	%	N	%	N	%
Gender						
Female	5	12.5	16	17.8	7	8.6
Male	35	87.5	74	82.2	74	91.4
Mean age	46.6		53.3		47.4	
Elite status						
Elite	33	82.5	42	46.7	47	58
Non-elite	7	17.5	48	53.3	34	42
Overseas Trainings						
Undergraduate	0	0	33	36.7	27	33.3
Graduate	12	30	22	24.4	24	29.6
Post-doctoral	12	30	13	14.4	40	49.4
N	40		90		81	

Data come from Ethics among Scientists in International Context (EASIC) study.

Percentages listed on this table do not represent the demographic characteristics of all physicists working in the three countries. They only represent the distribution of demographic characteristics among those who participated in the discussed study. Respondents’ demographic characteristics, including gender and age, came from their self-report during the interviews. Elite status comes from sampling stratifications based on research output. Finally, respondents’ overseas training was adapted from their self-report and cross-verified with information from the sampling frame

Table 2 provides a summary of this study’s interviewing methodologies following the consolidated criteria for reporting qualitative research (COREQ) (Tong et al., 2007).

**Table 2:** Methodological reporting following a revised COREQ checklist

Domain	Item	Description
Research team		
*Personal characteristics*		
1	Interviewer and credentials	Interviewers in the US and the UK: Both authors of this study and other members in the research team who have received a PhD degree in sociology or were training towards getting their PhD degree in sociology Interviewers in China: First author of this study, (native in mandarin and fluent in English), and a researcher who received a PhD degree in political science, (native in English and fluent in mandarin)
2	Gender	Interviews in the US and the UK: Alternating between male and female interviewers Interviews in China: Both male and female interviewers present in the settings
3	Experiences	All interviewers were trained through advanced qualitative methodology courses and received intensive training from the PI of this project, who is also the second author in this study
*Relationships with participants*		
4	Relationships established	No extensive prior relationships were established. Communications occurred primarily through emails for participant recruitment
5	Participant knowledge of the interviewer	Participants were informed about (1) the identity of the interviewer(s); (2) the goal of this study; (3) the protection of their confidentiality;(4) the potential benefits and risks associated with this study
6	Interviewer characteristics	Interviewers’ motivations, reasons, and interest in this study were shared with participants
*Theoretical framework*		
7	Methodological orientation and theory	A revised grounded theory approach: Researchers have developed some understandings of science ethics, which informed the design of the interview guide. But interview question wording and data analysis were also exploratory, enabling researchers to identify new themes
*Setting*		
8	Setting of data collection	Face-to-face interviews for respondents who were available during our scheduled trips Remote interviews for the respondents who were unavailable during the scheduled trips but wanted to participate in the study
9	Presence of non-participants	Not applicable. Most participants chose to conduct the interviews in their office with the presence of only the researchers and participant
*Data collection*		
10	Interview guides	Questions and prompts were designed by the PI of this study and provided by the authors. Pilot interviews were conducted with people in all three national contexts
11	Repeat interviews	No repeat interviews were conducted
12	Audio recording	Interviews in the US and the UK: With respondents’ permission, the interviews were audio recorded Interviews in China: Due to Chinese respondents’ concerns about confidentiality, to retain data validity, the interviews were not recorded. Instead, the first author of this study took transcript-like notes with respondents’ permission
13	Duration	The length of interviews ranges from 45 min to 1 h and 30 min
14	Data saturation	Participant recruitment stopped after hearing repeated themes in each national context, meaning that data saturation was reached
15	Transcripts returned	Transcripts were not returned to participants unless requested by the participants
*Analysis and findings*		
16	Data coders	The first author coded the data and the second author reviewed the data
17	Description of coding scheme	A summary of the coding schemes is available in Table 3
18	Participant checking	Participants did not provide feedback on data analyses
*Reporting*		
19	Quotation	Participants quotes were provided
20	Data and findings consistent	There was consistency between the data and findings
21	Clarity of major and minor themes	Major themes on respondents’ understanding science ethics as research, mentoring, and social responsibilities were discussed. Deviations from the major themes were analyzed as part of the cross-national differences

This table was adapted from the consolidated criteria for reporting qualitative studies (Tong et al., 2007). The items on sampling were not reported in the table as a comprehensive description of sampling strategy was reported in the data and methods section

In the case of China, the study concentrated on physics departments in three major university cities—Beijing, Shanghai, and Xi’an—that offer doctoral degrees in physics. The selection of universities in China was based on their inclusion in Project 985, an initiative to advance the development of world-class universities in China (Zhang et al., 2013). In the Chinese segment of the study, a total of 40 physicists participated, and Table 1 provides their demographic characteristics. Given the unique context and sensitivities in China, interviews were not recorded due to negative social connotations associated with recording what people say. Instead, transcription-like notes were taken during the interviews.

The interviews in China were conducted by two bilingual researchers: a male researcher served as the interviewer, while a female researcher worked as the note-taker. Participants from China were given the choice of being interviewed in Mandarin or English, with the majority of respondents (34) choosing Mandarin as their preferred language. This approach accommodated the linguistic preferences of the Chinese participants and contributed to a more comfortable and effective interview process within the Chinese scientific community.

The present study relied on physicists’ responses to the following interview question: “What does it mean to you to be a responsible researcher?” The word “responsible” was translated as *ze ren* in Mandarin. To ensure cross-national compatibility across interview guides, native and bilingual speakers reviewed the interview guide.

This interview question was analyzed because it directly inquired about the respondents’ perceptions of their responsibilities without providing further guidance or probing. It is important to note, however, that the responses may not necessarily encompass a comprehensive understanding of the scientists’ responsibilities. Furthermore, interview methods are susceptible to social desirability bias, where respondents may offer narratives that they perceive as socially acceptable in their social and institutional contexts (Bergen & Labonté, 2020). To mitigate this bias, following the approach of Bergen and Labonté (2020), the interviewers explained the study’s motivation, purpose, and procedures. In addition, when analyzing the data, the focus was on understanding why respondents provided specific narratives rather than assessing whether these narratives align with objective reality. This latter issue would require behavioral data rather than perception-based data.

The analysis was conducted in two rounds. In the first round, an inductive approach was employed, involving the reading of interview transcripts and the generation of coding schemes. In the second round, all transcripts were systematically reviewed to categorize the narratives, allowing for a comprehensive analysis of the interviews. This two-cycled coding approach aimed to ensure a rigorous and nuanced analysis of the data. Table 3 provides an overview of the codes created during the analyses, which allowed for a structured presentation of the findings.

**Table 3:** Summary of Coding Scheme

Cross-national themes	
Research	Honesty: Responsibility in conducting honest research Objectivity: Responsibility in making sure that findings are objective Robustness: Responsibility in checking the reliability and validity of research findings
Teaching and mentoring	Importance of teaching and mentoring: Teaching and mentoring as a set of responsibilities that is parallel to research Educating next-generation scientists: Preparing students to be future scientists. This preparation includes informing them of the research process, educating students on research ethics, and preparing students to be independent researchers
Social responsibility	Giving back: Giving back through research output, outreach, and other professional activities Research implications: Contributing to the society through research implications
UK and US-specific themes	
Social responsibility	Funding and social responsibility: Receiving public funding grants scientists an additional layer of social responsivity
China-specific themes	
Teaching and mentoring	Dilemma in teaching: Dilemma between the “modern” and “traditional” models in teaching
Social responsibility	Struggles in social responsibility: Struggles in discerning what society or sector of society scientists are responsible for

## Findings

### Conducting Honest Research

Respondents in China, the US, and the UK identified honesty as the most important characteristic of a responsible researcher.^1^ Narratives such as “being careful and honest about the work”^2^ and “being honest with my results and data”^3^ emerged consistently in all three countries, with some respondents referring to the definition provided by the Office of Research Integrity (2011). A Chinese physicist said, “[We] should not fabricate or plagiarize” and “you cannot change your results [even] if they are inconsistent with your expectations.”^4^

To some respondents, being honest means checking the robustness of their study. A US physicist^5^ said that scientists need to “check every calculation, making sure that it’s not an artifact of various assumptions.” For others, honest research is objective research. A US physicist^6^ said that physicists should “describe nature and not one’s emotions about nature.” If the research is “based [on] claims [that] are not reproducible,” it is “bad” and “subjective” science.

While acknowledging the importance of honesty in research, many respondents also saw a tension between honesty and working in a competitive community. A UK physicist^7^ said that being truthful means not making claims “that you haven’t verified fully” and waiting “until you are sure of your results before publishing.” Yet, publishing verifiable claims is easier said than done. He explained: “This is an issue in a very competitive environment where people will get something fairly minor and then oversell it, particularly [to] funding agencies.” It is, he believes, inevitable, that some scientists will use “buzz words” to exaggerate their results, which can in some instances sail close to deceitfulness.

One Chinese physicist^8^ with overseas training attributed the difficulty to maintaining full honesty in the evaluation system. Such a system, he said, encouraged academic scientists to focus only on short-term performance:

For many years, we have focused only on the number of publications, particularly the impact factor, citations, and h index (a method for evaluating the cumulative impact of an author’s scholarly output). All these things are created by foreigners. We imported these ideas into China, and Chinese universities treat them as important evaluative criteria for administration.

It is important to note that the pressure to publish in top-tier journals does not necessarily create difficulties for every scientist to do honest research. It is also important to note that the challenge, which involves balancing the demands of publishing with the imperative of conducting research with integrity, is not confined to China. What seems to set this Chinese scientist’s perception apart is the belief that foreign-created metrics for evaluating academic physicists’ productivity contribute to the difficulty in conducting honest research.

Another Chinese physicist^9^ shared a similar concern about the tensions between productivity and honesty:

Any kind of falsification, including intentional and unintentional, should not happen. [For example] the paper I’m currently working on I started in July 2012, and I [still] haven’t published the paper yet.

He said that he continually found errors in his work that many readers might not notice. Consequently, he invested a substantial amount of time on a single paper that remains to this day unpublished. He appears to believe that this prolonged timeline for publishing is a contributing factor in the frequent rejection of his grant applications.

While this physicist attributed his delayed publication pipeline to his devotion to conducting robust research, this perception does not necessarily reflect the reality of what might have happened. What is clear in this physicist’s narrative, however, is the perceived tension between the pressure to meet standardized evaluation criteria for faculty members and the responsibility of conducting honest research. This tension highlights the challenging balance between academic productivity and research integrity with which many scientists, including this physicist, wrestle.

Reflecting on their responsibilities as scientists, most physicists immediately emphasized honest research. The respondents also identified tensions between research honesty and survival pressures in a competitive academic community. This tension reflects the complex landscape that scientists navigate, where the need to publish, secure funding, and advance their careers can conflict with the ideals of research integrity.

Interestingly, while the interview question specifically asked about their perceptions of being responsible researchers, many physicists voluntarily brought up their mentoring and social responsibilities. This broader view of responsibility demonstrates that scientists are not focused solely on research but also consider their roles in nurturing the next generation of scientists and contributing to society as a whole. It underscores the multifaceted nature of the responsibilities that scientists acknowledge and uphold.

### Mentoring Junior Scientists

The physicists in this study generally see their mentoring responsibility as being integral to their responsibility as scientists. For instance, a US physicist^10^ said, “I have a responsibility to educate the students and postdocs I’m working with, and to help launch their careers.” She sees this responsibility as a parallel duty “to be honest in the analysis and presentation of data.” Her colleagues in the UK and China similarly said that mentoring is an important part of research. Indeed, UK-based physicists said that their “first and foremost responsibility”^11^ and “biggest responsibility”^12^ was as educators. One Chinese physicist^13^ said, “We need to be responsible to students, trying our best to push them to do their best in their research.”

Reflecting on what responsible mentorship means, participants in all three countries referred to the importance of nurturing their students’ interest in science. A UK physicist said that he “gives (students) a little bit more time to achieve goals and to produce research results.”^14^ A US physicist said that his “primary responsibility” as a teacher and mentor is “to do the very best we can at extracting beauty out of our understanding of nature.”^15^ A colleague in China said: “How can we stimulate students’ interest? When they figure out answers to [physics] questions, they will like physics and become interested in it.”^16^

Responsible mentorship also involves telling junior researchers what it means to be ethical scientists. As one US-based physicist said, “It’s not only teaching them science but teaching a culture—what being a scientist means.”^17^ Another physicist in the US shared that he “will tell them (students) to be extremely careful about fine-tuning their results,” especially when “looking at the data to predict comparisons [between it and your expectation].”^18^ A Chinese physicist said that “being a teacher, especially in China, not only involves conveying knowledge to students but also telling them how to be good people by acting as role models and informing them about moral issues.”^19^

Although most of our respondents said that responsible research entailed responsible mentoring, a context-specific narrative emerged from China-based interviews. Chinese physicists said that responsible mentors think carefully about whether they want to maintain hierarchical or egalitarian relationships with students—a consideration that did not appear in our interviews with US and UK physicists. A Chinese professor of physics^20^ said:

My perspective may be closer to the Chinese traditional perspective. I don’t think that students and teachers are equal. Teachers are there to teach students, not to listen to whatever demands students have. I oppose the way it is developing in the US—to assess the teacher’s performance entirely based on the students’ evaluation.

To underscore his point, he quoted a Chinese proverb—“It is only when teachers are respected that morals and knowledge can be respected” (*Zun Shi Zhong Dao*)—that highlights the importance of respecting teachers, ethics, and knowledge*.* In Mandarin, the word “teacher” can mean both mentor and instructor. Hence, it is unclear whether the respondent was referring to his role in the classroom, laboratory, or both. Nevertheless, he saw a tension between the “Chinese traditional perspective” and “the way it is developing in the US” in mentoring and chose to adopt the Chinese model.

The issue of navigating between two mentoring styles was also discussed in a conversation with a different Chinese professor.^21^ This respondent said that the professor-student relationship in China is complicated, with two ways of managing it: traditional and modern. Those who adhere to the traditional view believe that students should follow their professors, while those who support the modern view believe that students should be independent. He acknowledged that, due to his overseas experience, he preferred establishing “modern” relationships with his mentees.

It is not surprising that physicists in all three societies emphasized the significance of responsible mentoring as a crucial aspect of being responsible scientists. This shared perspective underscores the universal recognition of the role that mentoring plays in fostering a positive and ethical scientific community. However, what distinguishes Chinese physicists is the unique tension they experience in their mentoring roles. They see a need to choose between adhering to traditional hierarchical relationships with their students and embracing contemporary egalitarian ones. This tension reflects the cultural and societal dynamics at play in China, where traditional values may influence academic relationships and expectations. It reveals a nuanced interplay between Confucian cultural norms and modern scientific practices, which can have a substantial impact on how mentoring is perceived and practiced in the Chinese scientific community.

### Serving the Public

From the perspectives of academic physicists in China, the US, and the UK, an integral facet of fulfilling the role of a responsible scientist is engagement in social responsibility. For instance, a UK physicist^22^ emphasized the public nature of his university:

Working at a public institution, we do research, but we do so on behalf of the university or behalf of another funding body. [We also do research] on behalf of the science and technology facility centers and the research councils of the UK government. So, I have a responsibility towards all these bodies for the highest professionalism, and that encompasses a wide range of aspects and the way you come out with research, in the way you publicize that research, [and] in the way you also try to explain that research to the public and students.

For this scientist, there seems to be a clear connection between working in a publicly-funded institution and fulfilling his social responsibilities. Specifically, the receipt of public funding serves as *one of* the motivating factors for him to carry out his obligation to publicize his research “for the highest professionalism.” This connection between public funding and a commitment to transparently disseminate research findings underscores the sense of accountability that scientists in publicly-funded institutions often feel towards the broader public, who are ultimately the benefactors of their research and also its contributors.

A US physicist similarly underscored the “public” nature of his responsibilities. He said that working in a public university he is “a public servant” and “not very different from an elected official” because both “the federal government” and “the state” partially fund his work.^23^

Linking their social responsibility with public funding is common among US and UK physicists. One UK physicist said that “the thing that comes off the top of my head immediately is not to waste public money on something no good.”^24^ Likewise, a US physicist said that if the public sector “is paying for it (a research project),” then scientists “have a strong responsibility” to serve the public. This includes “making your results accessible to the public” and “communicating what you are doing to the public in non-technical language.”^25^ While it is common for respondents in the US and the UK to link their social responsibility with their reception of public funding, this does not mean that physicists in the two societies are solely responsible for the source of funding. Rather, their narratives suggest that receiving public funding offers them a greater sense of social responsibility.

Like their colleagues in the US and the UK, Chinese respondents acknowledged their responsibility to serve the public. However, what sets Chinese physicists apart is that they introduced an additional layer of consideration not seen in interviews with physicists in the US and the UK. Chinese physicists engaged in discussions about what constitutes the “public.” This suggests a distinct and nuanced perspective on their societal responsibilities.

The debate about defining the “public” reveals the complex relationship between cultural, social, and political factors in China, which can influence how scientists perceive and address their obligations to the broader society. It underscores the importance of considering cultural context when examining scientists’ perspectives on their social responsibilities and the diverse ways in which these responsibilities may be understood and enacted in different parts of the world.

For example, a Chinese physicist said that working at a public university meant that he must “complete assignments handed down by the government.”^26^ These assignments include “serving as peer reviewers for research projects [funded by the government], participating in conferences, and providing a project report,” all of which are part of his “social responsibility.” The society that he feels he has a responsibility to serve consists of local and national governments.

Another Chinese physicist^27^ said that academic scientists should serve the interests of humanity as a whole. Specifically, he believes that “scientists should be responsible not to one country, ethnicity, or party.” To illustrate his point, he differentiated between science and technology: “When we are doing technology, every country has some conservative aspects, including the US and China.” This “degree of conservatism,” he said, does not apply to the scientist’s public responsibilities. However, by “doing science” he believes that he has a responsibility to “serve humanity.” Neither respondent explicitly discussed how they would navigate conflicts between serving the government and serving humanity. Nor did they elaborate on how they would fulfill their responsibilities to humanity. Still, despite these omissions, a discernible pattern emerged: physicists in China appear to consider what “public” and what “society” they are serving.

## Discussion

The question of whether and how scientists’ ethical perspectives are globalized or context-specific remains a subject of ongoing inquiry (Stemerding et al., 2015). On the one hand, countless academic scientists are involved in extensive international collaborations (Leydesdorff & Wagner, 2008b), which might suggest the presence of a global science ethics framework that transcends national boundaries. On the other hand, the fact that these scientists live and work in different national contexts implies that their ethical perspectives might be influenced by the context-specific moral norms and values in their respective countries (X. Zhang & Sun, 2014).

This study, based on interviews with physicists in China, the US, and the UK, offers several novel insights into this question. By examining the ethical perspectives of scientists from three different national contexts, the study sheds light on the interplay between global and context-specific factors that shape scientists’ understanding of research ethics and societal responsibilities. It highlights the complex dynamics at play in the ethical landscape of the scientific community and how these dynamics are influenced by both global collaboration and unique national contexts.

A recap of the study’s findings indicates that physicists in all three countries cited three fundamental components in their responsibilities: research, mentoring, and social responsibility. Most of the respondents expressed a responsibility to conduct honest, robust, and verifiable research, to teach and mentor their students conscientiously, and to have a duty to the broader society. Cross-national differences were evident in how Chinese physicists articulated their mentoring and social responsibilities in comparison to their counterparts in the US and the UK. The Chinese physicists debated the merits of adopting either an egalitarian mentoring model, treating their mentees as peers, or a hierarchical model that emphasizes the authority of advisers.

Similarly, in terms of their social responsibility, physicists in China, the US, and the UK collectively uphold the belief that they bear a responsibility towards the public sectors that finance their research. However, a noteworthy divergence emerges among Chinese physicists, characterized by a certain ambivalence about interpreting the term “public.” This ambiguity concerning the specific definition of the “public” they should serve can be attributed to China’s relatively recent integration into the global scientific community. Its emergence as a significant new player in international science collaborations has introduced unique dynamics and considerations. These scientists may be wrestling with the evolving norms and expectations related to their societal responsibilities, and the debate about which public to prioritize may reflect the ongoing transformation of China’s scientific community in a global context (Leydesdorff & Wagner, 2008b).

To explain these findings, our study placed them in a theoretical framework of the scientists’ agency. In social sciences, agency is defined as the active involvement of actors within social structures and institutions, potentially leading to the replication or alteration of the structural or institutionalized milieu (Emirbayer & Mische, 1998). Caroline Whitbeck (1995) applied the concept of agency to science ethics and views scientists as moral agents, capable of creatively engaging with institutionalized ethical standards when forming their ethical perspectives.

Expanding on Whitbeck’s (1995) theorization of scientists as moral agents, this study further argues that scientists’ moral agency is most pronounced when they encounter tensions between different moral norms. In the global scientific community, such tensions can be more salient for the new players in international research collaborations (Leydesdorff & Wagner, 2008a). In this study, for China-based physicists, as relatively new participants in international research collaborations, these tensions arise from competing discourse between traditional Chinese ethics and science ethics from Western societies, such as the US and the UK.

Specifically, Confucian ethics encourage Chinese physicists to maintain hierarchical relationships with their students (Y. B. Zhang et al., 2005), while Western science ethics promotes egalitarian relationships. The ethical tensions also manifest in the realm of social responsibilities, where Chinese physicists contend with serving either humanity as a whole or primarily their own country. These ethical dilemmas prompt Chinese physicists to actively interpret, choose between, and justify their ethical norms. In contrast, physicists in the US and the UK, who have been shaped by science ethics primarily developed in Western societies, experience fewer potential tensions with a general science ethics. It is, however, essential to note that this comparison does *not* *mean* that physicists in the US and the UK do not have moral agency or have less moral agency than their Chinese counterparts. Instead, this cross-national comparison of physicists’ moral agency indicates that US and UK-based physicists’ *manifestation* of their moral agency may be less salient due to the fewer tensions that they experience from competing ethical frames.

In addition to the main findings, two supplementary observations emerged from this research. First, some physicists said that the pursuit of ethical research can conflict with their professional aspirations, a pattern also found in research conducted with life scientists in Denmark (Davies, 2019). This suggests that enhancing ethics in scientific communities may require a revision of the academic evaluation system. Second, physicists in all three countries perceive their responsibilities as teachers and mentors to be as important as their responsibilities as researchers. While they did not explicitly distinguish between verbal mentoring and role modeling, this raises interesting questions about how physicists integrate different forms of mentoring, which future studies could explore.

This research has several limitations. It focuses solely on the perspectives of academic physicists who participated in the study, and the findings may not be generalizable to physicists in other countries or scientists in different disciplines. Additionally, as qualitative research has inherent limitations, the study cannot establish a direct relationship between demographic characteristics and participants’ ethical perspectives.^28^ The majority of interviews were conducted between 2013 and 2014, and the articulation of research ethics by scientists may have evolved since then. Finally, the interview data reflects the respondents’ perceptions, which may not align with the real world. Nevertheless, the strength of the interview as a research method lies in its ability to identify why physicists develop specific perceptions in their own terms, complementing observation-based studies that capture behavioral data (Maxwell, 2004).

## Conclusion

Unlike previous studies focused on science ethics within a single national context (e.g., Ho & Oladinrin, 2019; Satalkar et al., 2016), this study has adopted a cross-national comparative perspective, providing a systematic understanding of the differences and similarities in scientists’ ethical perspectives. While the views of academic physicists in China, the US, and the UK converge on broad principles, such as the significance of honesty and objectivity in research, this study uncovered variations in their interpretations of scientists’ ethics in mentoring and service to society. Additionally, it offered insights into how academic scientists navigate the tensions arising from competing ethical and moral norms through their active interpretations of these norms.

 The research by Wagner and Leydesdorff (2009) highlights the significant involvement of numerous scientists in international collaborations. Nevertheless, it is crucial to recognize that these scientists maintain strong connections with their respective national contexts. From this vantage point, we can derive three overarching conclusions concerning cross-national science ethics.

First, this investigation underscored that scientists’ ethical perspectives are shaped not only by the widely accepted ethical and moral norms within the academic science community but also by the prevailing moral norms within their specific national contexts. For academic scientists engaged in international collaborations, an awareness of how and why their collaborators’ ethical perspectives may differ or align with their own can facilitate more seamless and productive collaborative endeavors.

Second, the findings of this study carry implications for science ethicists and policymakers. When formulating rules, processes, and procedures in the realm of science ethics, it is imperative to consider the perspectives and input of academic scientists, particularly those operating in countries with developing science infrastructures. By incorporating their voices, a more comprehensive and context-sensitive framework for science ethics can be established.

Third, although the specific insights into science ethics provided by the study’s respondents may not be directly transferable to scientists in other national contexts or different scientific disciplines, the theoretical argument put forth in this study emphasizes academic scientists’ own moral agency in actively interpreting and reconciling competing ethical frameworks between their nation and the global scientific community. This theoretical argument can be applied more broadly. Scholars researching science ethics can draw from this study’s findings to underscore the significance of analyzing how academic scientists in diverse national contexts comprehend, interpret, and reconcile conflicting ethical norms. Such an understanding can contribute to the development of more contextually informed ethical frameworks for the scientific community.

## Footnotes

1 In the US context, assistant professors are faculty members who have not received tenure, meaning that they have not received the job security of not being dismissed unless in extreme circumstances (Brown & Kurland, 1990). Associate professors, in the vast majority of the US-based universities, are mid-career researchers who have received tenure. Full professors are senior researchers. Academic ranking in the Chinese universities where we conducted research resemble the ranking in US-based universities. In the UK, lecturer is equivalent to assistant professor in the US, a senior lecturer is equivalent to associate professor in the US and reader or professor are equivalent to professor or name chaired professor respectively (Santos & Dang Van Phu, 2019).

2 US_012, research professor, male, interviewed on 03/26/2013.

3 UK_010, professor, male, interviewed on 09/24/2013.

4 CH_035, associate professor, male, interviewed on 03/14/2014.

5 US_007, professor, male, interviewed 03/25/2013.

6 US_012, research professor, male, interviewed 03/26/2013.

7 UK_007, professor, male, interviewed 09/19/2013.

8 CH_031, professor, male, interviewed 08/14/2013.

9 CH_023, associate professor, male, interviewed 12/20/2013.

10 US_008, associate professor, female, interviewed 03/25/2013.

11 UK_004, professor, male, interviewed 09/17/2013.

12 UK_021, senior lecturer, male, interviewed 10/02/2013.

13 CH_020, professor, male, interviewed 12/19/2013.

14 UK_060, professor, male, interviewed 03/21/2014.

15 US_026, professor, male, interviewed 04/29/2013.

16 CH_024, professor, female, interviewed 12/20/2013.

17 US_013, professor, male, interviewed 03/26/2013.

18 US_006, assistant professor, male, interviewed 03/22/2013.

19 CH_001, professor, male, interviewed 08/01/2013.

20 CH_014, professor, male, interviewed 08/14/2013.

21 CH_008, professor, male, interviewed 08/05/2013.

22 UK_029, reader, male, interviewed 10/14/2013.

23 US_036, associate, male, interviewed 05/03/2013.

24 UK_036, lecturer, male, interviewed 02/24/2014.

25 US_031, professor, male, interviewed 03/26/2013.

26 CH_010, professor, male, interviewed 08/07/2013.

27 CH_029, professor, male, interviewed 12/26/2013.

28 While this study does not investigate how the ethical perspectives of scientists relate to their demographic characteristics, it does not imply that such associations are absent. Indeed, prior research conducted by Ecklund and Di (2017) has indicated that there could be gender-based variations in how individuals approach workplace ethics.
